# Expression of pAkt affects p53 codon 72 polymorphism-based prediction of response to radiotherapy in nasopharyngeal carcinoma

**DOI:** 10.1186/1748-717X-8-117

**Published:** 2013-05-11

**Authors:** Xiaoxue Xie, Hui Wang, Hekun Jin, Shuyu Ouyang, Jumei Zhou, Jun Hu, Xuping Xi, Junming Luo, Yingying Zhang, Bingqiang Hu

**Affiliations:** 1 Department of Radiation and Oncology, Hunan Provincial Tumor Hospital & Affiliated Tumor Hospital of Xiangya Medical School, Central South University, Changsha 410013, China; 2 Department of Radiation and Oncology, Xianya Hospital, Central South University, Changsha China; 3 Department of Pathology, Hunan Provincial Tumor Hospital & Affiliated Tumor Hospital of Xiangya Medical School, Central South University, Changsha China

**Keywords:** Nasopharyngeal carcinoma, p53 codon 72 polymorphism, pAkt, Radiotherapy

## Abstract

**Background:**

Codon 72 (Arg/Pro), the most frequently studied single nucleotide polymorphism (SNP) of p53 to date, is associated with the ability of the gene to induce cell apoptosis. The PI3K/Akt pathway plays an essential role in the transcriptional activation function of p53, and is an important factor in radiotherapy resistance. The present study was designed to evaluate the prediction of response to radiotherapy based on p53 codon 72 SNP and pAkt expression in biopsy specimens of locoregional nasopharyngeal carcinoma (NPC) before treatment.

**Materials and methods:**

In total, 75 consecutive patients with locoregional NPC were enrolled. The p53 codon 72 SNP was identified from retrospectively collected paraffin-embedded biopsy specimens using Sanger sequencing. Expression patterns of p53, p21, 14-3-3σ, and pAkt proteins were investigated using immunohistochemical analyses. The effects of genetic polymorphisms and protein expression on progression-free survival (PFS) were evaluated using the Cox proportional hazards model, Kaplan–Meier method, and log-rank test.

**Results:**

The p53 codon 72 Pro/Pro carriers showed lower risk of disease progression (local recurrence and distant metastases) (HR: 0.300; 95% CI: 0.092–0.983; *p*=0.047). However, this association between the p53 codon 72 polymorphism and PFS was not significant in the pAkt-positive subgroup. No association was observed between protein expression of p53, p21 or 14-3-3σ and p53 codon72 polymorphisms. Notably, positive expression of p53 protein appeared to be correlated with poorer PFS among patients diagnosed as local regional lymph node metastasis (N+) before treatment (*p*=0.032).

**Conclusions:**

The p53 codon 72 Pro/Pro genotype may be an effective independent prognostic marker for better outcome in patients with locoregional NPC. Based on the current findings, we hypothesize that pAkt weakens the predictive value of p53 codon 72 SNP in NPC. A combination of positive p53 protein expression and local regional lymph node metastasis may additionally be predictive of high risk of disease progression.

## Introduction

Nasopharyngeal carcinoma (NPC) is an epithelial malignancy with an annual incidence of 15 to 50 cases per 100,000 individuals in Southern China [1]. The prognosis of patients with NPC in the early stages is generally favorable, although the incidence of relapse remains high for locoregionally advanced NPC [2,3]. Radiotherapy remains the primary mode of treatment for locoregional NPC. In the present study, we focused on evaluating the predictive value of the p53 pathway in outcomes of patients with locoregional NPC in response to radiotherapy. The p53 gene is thought to be central in mediating response to commonly used cancer treatments, such as radiotherapy. Codon 72 (Arg/Pro) is the most frequently studied SNP in the p53 gene to date. The amino acid encoded by codon 72 resides in a poly proline region located between the transactivation and DNA binding domains. This proline-rich region has been shown to be critical for p53 function, particularly its ability to induce apoptosis. Numerous studies have provided evidence that the two different isoforms encoded by p53 codon 72 SNP are not functionally equivalent [4-8]. In a number of cancer types, including lung and breast, patients with the 72Pro-p53 allele appear to be more susceptible to tumor development [9-12]. However, there is no consensus regarding the correlation between treatment susceptibility and p53 polymorphism at codon 72 in cancers. For example, Vannini *et al*. [13] reported a significantly shorter time of disease progression and overall survival in metastatic breast cancer patients homozygous for Arg, compared to those with heterozygous Arg/Pro tumors. Conversely, Kim *et al*. [14] reported that Arg/Pro and Pro/Pro genotypes of TP53 codon 72 are significantly correlated with a lower response rate to combination chemotherapy, compared to the Arg/Arg genotype in advanced gastric cancer. Results from a meta-analysis [15] showed that homozygote Arg/Arg genotype is more significantly associated with decreased risk of NPC, compared with the Pro/Pro genotype. However, the precise relationship between the TP53 codon 72 polymorphism and clinical outcomes of NPC remains to be established.

The phosphatidylinositol-3-kinase (PI3K)/protein kinase B (Akt) pathway plays an essential role in the transcriptional activation function of p53 and consequent modulation of cell fate. Notably, EGFR-independent activation of the PI3K/Akt pathway, a common occurrence, is an important factor in radiotherapy resistance [16]. Akt is a downstream effector of PI3kinase in NPC possibly associated with aggressive tumor behavior and poor survival in patients with nasopharyngeal carcinoma [17]. The existing paradigm about the nature of interactions between the PI3-kinase and p53 pathways is antagonistic, whereby pAkt inhibits p53 function in a Mdm2-dependent manner [18-20]. Under conditions where the apoptotic activity of p53 prevails, it is conceivable that the destruction of pAkt plays a role in accelerating the apoptotic process.

The relationship between p53 activation and cell fate regulation is extremely complex [21]. Boehme *et al*. [22] proposed a signaling cascade involving activation of DNA-PK and Akt/PKB for the regulation of p53 in response to ionizing radiation. Suvasini *et al*. [23] also reported an essential function of PI3-kinase and its downstream effectors, Akt/PKB-mTOR, in activating p53-mediated transcription during DNA damage. In the cell membrane, Akt is activated through phosphorylation at serine and threonine residues, and modulates the expression of several genes involved in suppression of apoptosis and cell cycle progression. The mechanism by which the DNA damage-induced PI3K/Akt pathway alters p53 phosphorylation at critical residues is not clear at present. However, reduced Lys382 acetylation in LY294002-treated cells may be explained by the finding that p300, which acetylates p53, is activated by Akt-mediated phosphorylation [24]. Thus, the classical identification of the ‘pro-survival’ PI3K/Akt and ‘pro-apoptotic’ p53 pathways adds another layer of complexity to the outcome of NPC.

The present study was designed to evaluate prediction of response to radiotherapy on the basis of p53 codon 72 SNP and p53 pathway protein expression in locoregional nasopharyngeal carcinoma (NPC) biopsy specimens before treatment. To our knowledge, this is the first study to focus on assessing the combined prognostic effects of p53 codon 72 SNP and pAkt expression.

## Materials and methods

### Patient selection

Between January 2008 and October 2009, 75 consecutive patients with locoregional NPC at the Department of Radiation and Oncology of the Hunan provincial tumor hospital were enrolled retrospectively in this study. Patients with proven biopsy and previously untreated NPC with American Joint Committee on Cancer (AJCC)/International Union Against Cancer (UICC) stages II, III and IV (A-B) were eligible for study. Other criteria included ages greater than 18 years, Han Chinese ethnicity, and an Eastern Cooperative Oncology Group performance status of 0 or 1. The exclusion criteria included presence of distant metastasis and other concomitant malignant disease. The study was approved by the Clinical Research Ethics Committee of the Hunan Province Cancer Center, and written informed consent was obtained from all patients. Patient characteristics are summarized in Table 1. In total, 57 male and 18 female patients (male-to-female ratio of 3.2:1) with a median age of 45 years (range, 22–72 years) were enrolled. All patients were diagnosed as World Health Organization (WHO) Grade 2–3 NPC. Among our patient population, 16 had stage II, 44 had stage III, and 15 had stage IV (A-B) disease.

**Table 1:** Patient demographics and treatment characteristics

**Characteristics**	**Number of patients (%)**
Age, y	22-72
Median age	45
Gender	
Male	57(76.0)
Female	18(24.0)
Overall stage (AJCC ) ^*^	
II	16(21.3)
III	44(58.7)
IVa-b	15(20.0)
T classification^*^	
T1-2	42(56.0)
T3-4	33(44.0)
N classification^*^	
N0	16(21.3)
N1-3	59(78.7)
Concurrent chemotherapy	
No	36(48.0)
Yes	39(52.0)
Adjuvant chemotherapy	
No	35(46.7)
Yes	40(53.3)

^*^American Joint Committee on Cancer/International Union Against Cancer staging system.

### Pretreatment evaluation

All patients were evaluated using complete physical examination, fiberoptic nasopharyngoscopy, MRI of the head and neck, chest X-ray, abdominal imaging with ultrasound, and bone scan.

### Treatment

Megavoltage photons (6 MV) were used to treat primary tumor and neck lymph nodes. Radiotherapy was administered five times a week at a dose of 2 Gy/d. The accumulated radiation dose to the primary tumor was 68 to 72 Gy, 60 to 62 Gy to the involved areas of the neck, and 50 Gy to uninvolved areas. Concurrent chemoradiotherapy, specifically, DDP (100 mg/m ^2^) on days 1, 22, and 43 during radiotherapy, was administered to 39 patients.

### End points

The primary end point for the study was progression-free survival (PFS), defined as the time from the day of enrollment to the date of first documentation of relapse categorized as locoregional (primary site or regional nodes) failure, distant metastases or last follow-up visit.

### Sample preparation, DNA extraction and quantification of DNA yield

From each sample, eight 10 μm tissue sections were deparaffinized using xylene and ethanol. Specimens were digested with proteinase K for 24 h at 55^C^. Subsequently, the enzyme was inactivated by boiling the samples for 10 min prior to mixing with absolute ethyl alcohol. After purification with ion-exchange columns, genomic DNA was stored at -20^C^ before use.

### DNA amplification and genotyping procedures

Nested PCR method was used to amplify specific fragments. The PCR reaction was performed in three steps: 5 min at 95^C^, followed by 35 cycles of 30 s at 95^C^, 30 s at 55^C^, and 40 s at 72^C^, and finally, 10 min at 72^C^. Prior to Sanger sequencing, amplified products were identified by electrophoresis using BigDye Terminator v3.1 chemistry (Life Technologies, Carlsbad, CA) on an Applied Biosystems 3130XL Genetic Analyzer. All PCR reactions and sequencing analyses were performed twice to confirm the results.

### Immunohistochemical studies

**Figure 1:**
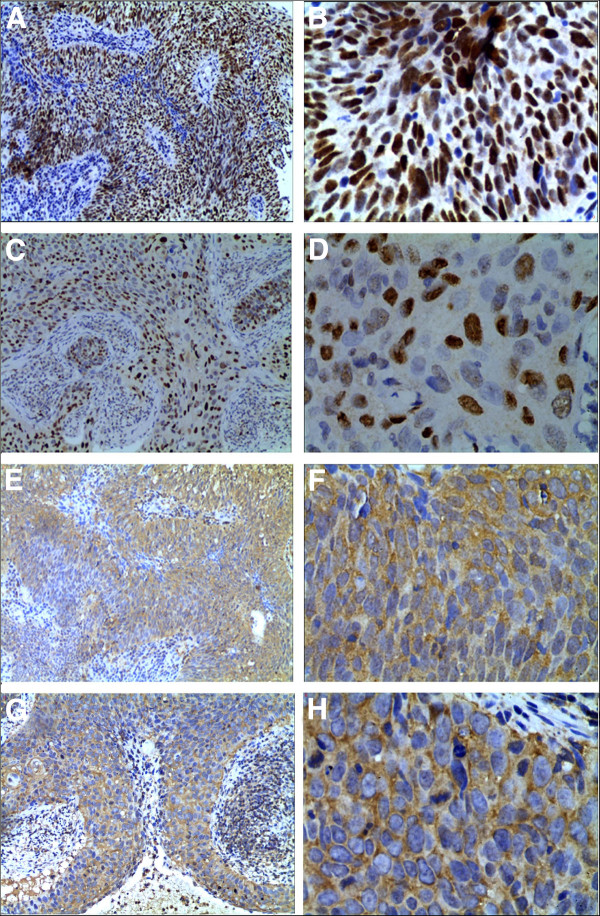
**Representative results of p53**, **p21**, **pAkt**, **and 14**-**3**-**3σ imunohistochemistry in NPC tissues.** Brown nuclear staining of tumor cells for p53 shown in **A** (× 100) and **B** (× 400), and for p21 in **C** (× 100) and **D** (× 400), and brown cytoplasm staining of tumor cells for pAkt shown in **E** (× 100) and **F** (× 400) and for 14-3-3σ in **G** (× 100) and **H** (× 400).

Immunohistochemistry (IHC) was performed on sections of 75 NPC tissues using standard techniques. Tissue sections were deparaffinized in xylene, rehydrated in a graded ethanol series, and treated with an antigen retrieval solution (10 mmol/L sodium citrate buffer, pH 6.0). Next, sections were incubated with mouse anti-human p53 antibody (1:200 dilution) overnight at 4^C^ and 1:1,000 diluted biotinylated secondary antibody, followed by avidin-biotin peroxidase complex (DAKO, Carpinteria, CA), according to the manufacturer’s instructions. Finally, tissue sections were treated with 3’, 3’-diaminobenzidine (Sigma) until the development of brown color, and counterstained with Harris modified hematoxylin. In negative controls, primary antibodies were omitted. The procedure for staining pAkt, p21, and 14-3-3σ was similar to that for p53 IHC staining, except that the anti-p53 antibody was replaced with anti-pAkt (Thr308)(Santa Cruz Biotechnology, Inc.), anti-p21(ZSGB-BIO), and anti-14-3-3σ(Abcam) antibodies, respectively. Sections were blindly evaluated by two investigators in an effort to provide a consensus on staining patterns. A semiquantitative scoring criterion for immunohistochemistry was used in which both staining intensity and positive areas were recorded according to the method of Hara *et al*. [25]. At least 10 high-power fields were selected randomly, and >1,000 cells counted for each section. Staining intensity was graded on the following scale: 0: no staining, 1+: mild staining, 2+: moderate staining, and 3+: intense staining. The area of staining was scored as follows: 0: no staining in any microscopic field, 1+: <30% of tissue stained positive, 2+: between 30% and 60% stained positive, and 3+: >60% stained positive. The minimum score, when summed (extension+ intensity), was 0, and the maximum score was 6. For 14-3-3σ and pAkt, a combined staining score (extension+ intensity) of ≤ 3 was considered negative (low staining), and that between 4 and 6 considered positive. For p53 and p21, a combined staining score (extension+ intensity) of ≤ 4 was considered negative (low staining), and that between 5 and 6 considered positive. Representative results of p53, p21, pAkt, and 14-3-3σ immunohistochemistry are shown in Figure 1.

### Follow-up studies

The follow-up analysis was completed on October 31, 2012, with a median follow-up of 25 (range, 5–46) months. After completion of treatment, patients were followed up at least every three months during the first years and every six months thereafter until disease progression (recurrence or distant metastases). All local recurrences were diagnosed based on fiberoptic endoscopy and biopsy and/or MRI of the nasopharynx and the skull base showing progressive bone erosion and/or soft tissue swelling. Regional recurrences were diagnosed by clinical examination of the neck and, in doubtful cases, fine needle aspiration or MRI of the neck. Distant metastases were diagnosed on the basis of clinical symptoms, physical examination and imaging methods, including chest radiography, abdominal ultrasound, whole body bone scan, CT scan, and MRI. During follow-up, 22 (29.3%) patients had locoregional relapse and 21 (28.0%) had distant metastasis. No patients died during follow-up. The 3-year PFS rate was 42.7% (32/75).

### Statistical analysis

The correlation between IHC results and p53 codon 72 genotypes was evaluated using Pearson χ^2^ test. The Hardy–Weinberg equilibrium was tested using a goodness-of-fit χ^2^ test with one degree of freedom. The Kaplan–Meier method was adopted to estimate survival curves, and the log-rank test used to compare patient survival times between subgroups. Multivariate analyses using Cox regression were employed to assess the importance of clinical variables, with adjustment for age, gender, T classification, N classification, and concurrent chemotherapy. Analyses were carried out using the statistical software package SPSS 17.0 (SPSS). All statistical tests were two-sided, and a value of *p* < 0.05 considered statistically significant.

## Results

**Figure 2:**
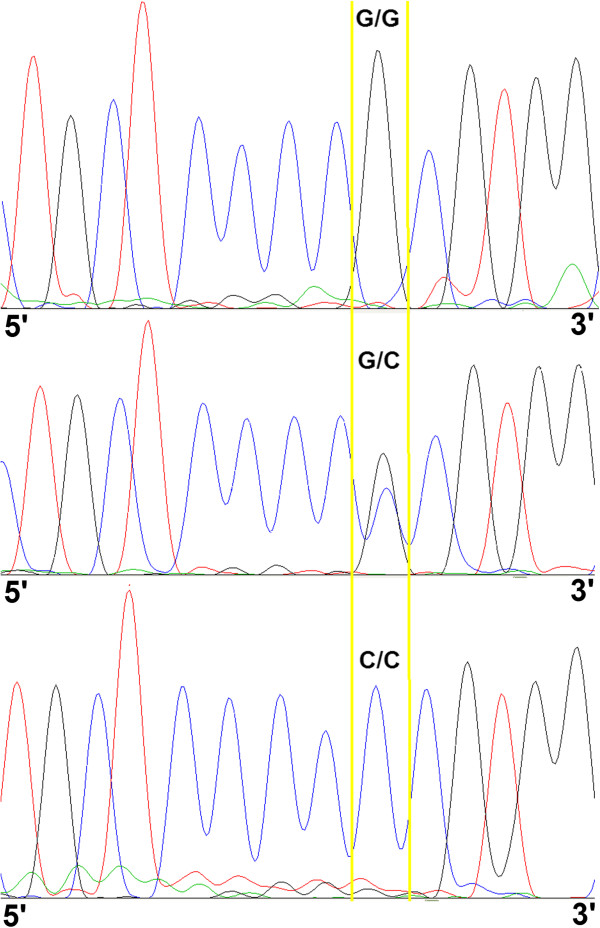
**Typical raw data obtained using Sanger sequencing instruments for p53 codon 72 (G>C) polymorphisms.** Note: The area between the yellow lines indicates the resulting genotypes; p53 codon72 G is equivalent to Arg and C is equivalent to Pro.

The p53 codon72 polymorphism was clearly distinguished via Sanger sequencing. Typical sequencing peak diagrams are depicted in Figure 2. The frequency of the Arg allele was 0.23, fulfilling the Hardy-Weinberg distribution. We observed no significant association between the p53 codon72 SNP and clinical variables (Table 2). Expression patterns of p53, p21, and 14-3-3σ were not affected by p53 codon72 polymorphisms (Table 3).

**Table 2:** **Correlation of p53 codon 72 genotypes with clinical variables** (**Chi**-**square analysis**)

**Arg/Arg**		**Arg**/**Pro**		**Pro**/**Pro**		***p***
T1-2	T3-4	T1-2	T3-4cp	T1-2	T3-4	
17(40.48%)	16(48.48%)	19(45.24%)	11(33.33%)	6(14.29%)	6(18.18%)	0.810
N0	N+	N0	N+	N0	N+	
8(50%)	25(42.37%)	5(31.25%)	25(42.37%)	3(18.75%)	9(15.25%)	0.842
Stage II	Stage III-IV	Stage II	Stage III-IV	Stage II	Stage III-IV	
7(43.75%)	26(44.7%)	7(43.75%)	23(38.98%)	2(12.5%)	10(16.95%)	0.842

**Table 3:** **Association between p53 codon 72 genotypes and p53**/**p21**/**14**-**3**-**3σ protein expression** (**Chi**-**square analysis**)

		**p53 codon 72 genotypes**			
**Protein expression**		**Arg/Arg**	**Arg/Pro**	**Pro/Pro**	***p***
p53	Positive	20	16	6	0.423
	Negative	12	13	6	
p21	Positive	8	5	3	0.748
	Negative	23	25	9	
14-3-3σ	Positive	18	17	3	0.100
	Negative	13	13	9	

**Figure 3:**
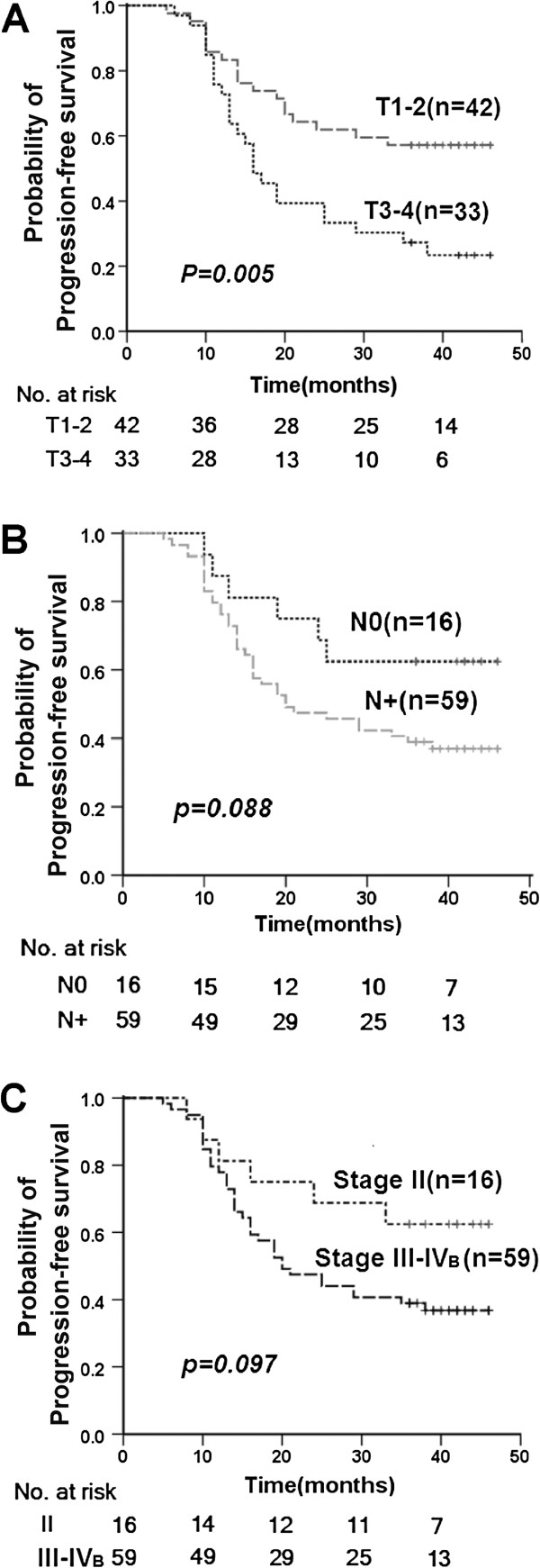
**Kaplan-Meier PFS curves according to clinic stages.** Kaplan-Meier PFS curves according to T classification **(A)**, N classification **(B)** and overall stage **(C)**.

For clinical variables, only T classification was associated significantly with the time to disease progression (Figure 3) (*p*=0.005). However, both T (HR: 2.683; 95% CI: 1.430–5.032; ***p*=0.002)** and N (HR: 1.778; 95% CI: 1.196–2.644; ***p*=0.004**) classifications were significantly correlated with 3-year PFS rates after adjustment for age (<45 and ≥45 years), gender (male and female), and concurrent chemotherapy (yes or no) (Table 4). Hazard ratios (HR) for disease progression (distant metastases and local recurrence) were significantly lower for Pro/Pro p53 codon 72 carriers after adjustment (HR: 0.300; 95% CI: 0.092–0.983; ***p*=0.047**) (Table 4). However, no overall association was evident between p53, p21 or 14-3-3σ protein expression and PFS (Table 4).

**Table 4:** **Multivariate analysis of progress**-**free survival** (**PFS**) **using the Cox proportional hazards model**

**Variables**	**Subgroups**	**HR**	**95%CI**	***p***
T classification	T3-4 vs. T1-2	2.683	1.430-5.032	**0.002**
N classification	N+ vs. N0	1.778	1.196-2.644	**0.004**
p53 codon 72 genotype	C/C vs. G/C+G/G	0.300	0.092-0.983	**0.047**
p53 protein	Positive vs. Negative	1.714	0.893-3.291	0.105
pAkt protein	Positive vs. Negative	1.025	0.542-1.938	0.939
p21 protein	Positive vs. Negative	0.745	0.338-1.641	0.465
14-3-3σ protein	Positive vs. Negative	1.440	0.764-2.711	0.259

Abbreviations: *HR*, hazard ratio; *CI*, confidence interval.

Note: p53 codon72 G is equivalent to Arg and C is equivalent to Pro.

**Figure 4:**
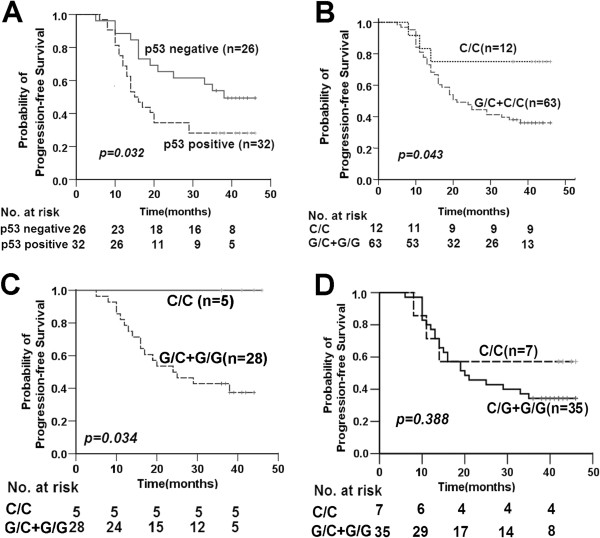
**Kaplan-Meier PFS curves according to P53.** Kaplan-Meier PFS curves according to protein expression of p53 (positive vs. negative) among patients diagnosed as local regional lymph node metastasis **(A)**; Kaplan-Meier PFS curves according to dominant model of p53 codon 72 SNPs (C/C vs. G/C+G/G) **(B)**; Kaplan-Meier PFS curves according to dominant model of p53 codon 72 SNPs (C/C vs. G/C+G/G) in the pAkt-negative **(C)** and pAkt-positive subgroups **(D)**. Note: p53 codon72 G is equivalent to Arg and C is equivalent to Pro.

As shown in Figure 4A, positive p53 protein expression was correlated with poorer PFS in a subgroup of patients diagnosed with lymph node metastasis (N+) before initial treatment (*p*=0.032). Kaplan-Meier PFS curves were obtained according to p53 codon 72 SNP and p53 expression, respectively (Figure 4). Patients carrying the Pro/Pro p53 codon 72 SNP displayed a longer time to disease progression than those with other polymorphic variants (*p*=0.043, Figure 4B). This result was significant in the pAkt-negative (*p*=0.034, Figure 4C), but not the pAkt-positive subgroup (*p*=0.388, Figure 4D).

Results of analysis of the combined effects of the p53 codon72 polymorphism and pAkt protein expression on risk of disease progression (local recurrence and distant metastases) are shown in Table 5. According to log-rank analysis, subjects carrying the p53 codon 72 Pro/Pro genotype displayed significantly longer time to disease progression, compared to those with other genotypes, in the pAkt-negative subgroup **(*p*=0.034).** However, this result was not significant upon assessment with the Cox proportional hazards model (*p*=0.191). Moreover, no significant association between the p53 codon72 polymorphism and PFS was observed in the pAkt-positive subgroup.

**Table 5:** **Log**-**rank and proportional hazard analysis** (**Cox method**) **of progression**-**free survival** (**PFS**) **in relation to p53 codon 72 genotypes and pAkt protein expression**

**p53 codon 72 polymorphism**	**pAkt protein expression**	**Log**-**rank analysis**		**Cox**-**regression**		
		**Mean survival time**(**months**)	**p**	**HR**	**95% CI**	***p***
G/G +G/C	Positive	24.66±2.38	—	1	reference	—
C/C	Positive	29.86±6.72	0.388	0.626	0.187-2.096	0.447
G/G +G/C	Negative	25.79±2.48	—	1	reference	—
C/C	Negative	40.60±2.04	**0.034**	0.034	0.00-5.394	0.191

Note: p53 codon 72 G is equivalent to Arg and C is equivalent to Pro.

## Discussion

In the present study, patients carrying p53 codon 72 Pro/Pro displayed better PFS than those with other polymorphisms. Multivariate analysis of the three-year PFS rate with both Cox proportional hazards model and log-rank analysis of time to disease progression suggested that the p53 codon72 polymorphism is an independent predictor of response to radiotherapy in nasopharyngeal carcinoma.

Pro72 and Arg72 variants are reported to differ in terms of functional activity. Specifically, the Pro72 variant possesses enhanced ability to transactivate p21 and induce growth arrest [4,6,26,27], while the Arg72 variant demonstrates superior mitochondrial localization in tumor cell lines [5]. Interestingly, Dumont and co-workers showed that the Arg72 variant is more efficient than the Pro72 variant in inducing apoptosis. One possible mechanism underlying this greater efficiency is enhanced localization of the Arg72 variant to mitochondria [5]. In contrast, our results suggest that the p53 codon72 Pro/Pro genotype is associated with improved PFS in locoregional NPC patients. A recent study showed binding of the p53 homolog, p73, to specific tumor-derived mutant forms of p53, which is influenced by the codon 72 polymorphism. It was proposed that several common mutants of mtp53-codon72-Arg bind with greater affinity to p73, thereby inhibiting its ability to induce apoptosis [28-31]. However, the p53 gene is rarely mutated in nasopharyngeal carcinoma [32]. Additionally, the Arg72 variant has been shown to be more susceptible to degradation by the human papillomavirus (HPV) 18 E6 protein [33]. Approximately 15–20% of head-and-neck squamous cell carcinomas (HNSCC) may be linked to HPV infection [34-36]. Accordingly, the effect of HPV on response to radiotherapy of Arg72 variant carriers in NPC requires further study. Other mechanisms should also be considered. For example, Vannini *et al*. [13] provided a molecular explanation for association of the Arg allele with tumor aggressiveness and treatment resistance in advanced breast cancer. The group demonstrated that lower cell death is induced under hypoxia upon transfection of the Arg allele, compared to Pro allele *in vitro*, which was explained by the finding that the Arg allele upregulates BCRP-I, a hypoxia response gene, that increases treatment resistance. While the precise molecular mechanisms underlying these differences in apoptosis merit further study, these observations suggest that determination of the patient genotype at p53 codon 72 may aid in identifying patient populations with poorer response to radiotherapy.

The prognostic value of p53 protein expression in NPC has been investigated, but the results obtained to date are controversial. The groups of Faccioli [37], Genc [38], Shi [39], and Tsai [40] reported that p53 overexpression is not correlated with various clinical parameters and overall survival. However, Ma *et al*. [41] suggested that overexpression of p53 protein is related to poorer disease-free survival and a trend towards shorter time to progression, but not overall survival. In contrast, Masuda *et al*. [42] reported that p53 overexpression is associated with lower overall survival. Moreover, Wang and co-workers [43] demonstrated a significant association of p53 protein overexpression with locoregional recurrence of patients with NPC, but no impact on the 5-year survival rate. The present study also focused on p53 protein as a potential predictive marker of response to radiotherapy for locoregional NPC. Notably, expression of p53 protein was not affected by p53 codon 72 polymorphisms (Table 3). Moreover, no significant association was evident between p53 protein expression and PFS (Table 4). Nevertheless, patients expressing p53 showed poorer PFS among those diagnosed with locoregionally lymph node metastasis (N+) before treatment (*p*=0.032) (Figure 4C). Our data indicate that a combination of positive p53 protein expression and local regional lymph node metastasis can be effectively used to predict poorer outcomes.

P21/waf1 has been described as a wild-type p53-inducible protein involved in inhibition of cell cycle progression through binding to cyclin/cyclin-dependent-kinase (CDK) complex. Earlier studies have provided evidence that after DNA damage, p53 enhances p21/waf1 transcription, which, in turn, inhibits the formation of cyclin-CDK complexes, blocking progression along the cell cycle in the G1-S transition stage [44]. 14-3-3σ, a downstream target of p53, is upregulated in response to DNA damage, and acts as a negative regulator of cell cycle G2-M phase checkpoint. Moreover, 14-3-3σ interacts with p53 and positively potentiates its activity [45]. Yang *et al*. [46] found that overexpression of 14-3-3σ in NPC cells inhibits cell growth, sensitizes cells to apoptosis, counteracts Akt oncogenic signaling, and reduces tumorigenicity in nude mice. The function of 14-3-3σ as a potential tumor suppressor gene in NPC was similarly confirmed by Yi *et al*. [47]. Accordingly, we attempted to determine whether the predictive effect of p53 codon 72 polymorphism is correlated with p21 and 14-3-3σ expression. In our experiments, p21 and 14-3-3σ protein levels were not affected by p53 codon 72 polymorphisms. Based on this finding, we propose that the predictive value of p53 codon72 in response to radiotherapy for NPC is not associated with the regulation of downstream proteins, p21 and 14-3-3σ.

The PI3K/Akt pathway plays an essential role in the transcriptional activation functions of p53, thereby modulating cell fate decisions. Retrospective studies by Schuurbiers *et al*. [48] showed that pAkt expression is an independent prognostic indicator of clinical outcomes of lung cancer. Yip *et al*. [17] reported that Akt is activated in 42.2% and 35.9% NPC cases, based on pAkt (Thr308) and pAkt (Ser473) immunoreactivity, respectively. The group further demonstrated that pAkt promotes cell proliferation and survival and is critical in NPC pathogenesis. In the present study, positive expression of pAkt (Thr308) protein was detected in 48.53% (33/72) of the subjects. No significant association between pAkt and PFS was observed. However, pAkt protein expression appeared to affect the relationship between p53 codon72 polymorphisms and outcomes in NPC patients. Subjects carrying the p53 codon72 Pro/Pro genotype showed longer time to disease progression, compared to patients with other genotypes (*p*=0.043, Figure 4A). This result was significant among subgroups with negative pAkt protein expression (*p*=0.034, Figure 4B), while no significant association between p53 codon 72 polymorphisms and PFS was found in the pAkt-positive subgroup. According to Ogawara *et al*., both Akt expression and serum treatment increase MDM2 ubiquitination of p53 [20]. It has been reported that p53 phosphorylated at Ser-20 escapes degradation by Mdm2, leading to stabilization of the protein [49]. Ozeki *et al*. confirmed that Mdm2-mediated degradation of 72Arg-p53 is diminished, compared to 72Pro-p53, which is at least partly attributable to higher levels of Ser-20 phosphorylation in p53-72R [50]. One possibility is that 72Pro-p53 is more easily down regulated via activation of the PI3K/Akt pathway, compared to 72Arg-p53. Therefore, the PI3K-Akt pathway may contribute to cell fate determination via selective inhibition of 72Pro-p53 accumulation. As a result, the p53 codon72 Pro/Pro carriers show no superiority in terms of response to radiotherapy in the pAkt-positive subgroup.

Our data collectively suggest that the p53 codon72 Pro/Pro genotype of the p53 gene in locoregional NPC is a useful prognostic marker for identifying the patient populations that respond poorly to radiotherapy, particularly in those negative for pAkt expression.

## Conclusions

We have shown that the p53 codon72 Pro/Pro genotype presents an independent predictor of better outcome in locoregional NPC patients. Positive expression of pAkt appears to act as a suppressor of this predictive function of the p53 codon72 Pro/Pro genotype. We observed no involvement of p21 and 14-3-3σ expression in the mechanism underlying the predictive value of p53 codon72 in response to radiotherapy in NPC. In addition, our results indicate that a combination of positive p53 protein expression and local regional lymph node metastasis may effectively act as a positive predictor of high risk of disease progression.

### Grant support

Science Foundation for Post Doctorate Research from the China Hunan Provincial Science and Technology Department (No.: 2012RS4011).

## Competing interests

The authors declare that they have no competing interests.

## Authors’ contributions

XX, HW, BH, and JL carried out the projects design and paper writing. HJ, SO, and XX carried out the collection of clinic data. XX, HW, JH carried out the immunohistochemical studies. XX, HW, JZ and YZ carried out the DNA amplification and genotyping. All authors read and approved the final manuscript.

## References

[B1] Min HQ Gun GY Nasopharyngeal carcinoma Epidemiology 1996 Beijing, China: People's Medical Press 280 285

[B2] Huncharek M Kupelnick B Combined chemoradiation versus radiation therapy alone in locally advanced nasopharyngeal carcinoma:results of ameta-analysis of 1,528 patients from six randomized trials Am J Clin Oncol 2002 25 219 223 10.1097/00000421-200206000-00002 12040275

[B3] Langendijk JA Leemans CR Buter J Berkhof J Slotman BJ The additional value of chemotherapy to radiotherapy in locally advanced nasopharyngeal carcinoma: a meta-analysis of the published literature J Clin Oncol 2004 22 4604 4612 10.1200/JCO.2004.10.074 15542811

[B4] Thomas M Kalita A Labrecque S Pim D Banks L Matlashewski G Two polymorphic variants of wild-type p53 differ biochemically and biologically Mol Cell Biol 1999 19 1092 1100 9891044 10.1128/mcb.19.2.1092 PMC116039

[B5] Dumont P Leu J Della Pietra AC 3rd George DL Murphy M The codon 72 polymorphic variants of p53 have markedly different apoptotic potential Nat Genet 2003 33 357 365 10.1038/ng1093 12567188

[B6] Sullivan A Syed N Gasco M Bergamaschi D Trigiante G Attard M Hiller L Farrell PJ Smith P Lu X Crook T Polymorphism in wild-type p53 modulates response to chemotherapy in vitro and in vivo Oncogene 2004 23 3328 3337 10.1038/sj.onc.1207428 15077186

[B7] Pim D Banks L p53 polymorphic variants at codon 72 exert different effects on cell cycle progression Int J Cancer 2004 108 196 199 10.1002/ijc.11548 14639602

[B8] Bergamaschi D Samuels Y Sullivan A Zvelebil M Breyssens H Bisso A Del Sal G Syed N Smith P Gasco M Crook T Lu X iASPP preferentially binds p53 proline-rich region and modulates apoptotic function of codon 72-polymorphic p53 Nat Genet 2006 38 1133 1141 10.1038/ng1879 16964264

[B9] Fan R Wu MT Miller D Wain JC Kelsey KT Iencke JK Christiani DC The p53 codon 72 polymorphism and lung cancer risk Cancer Epidemiol Biomarkers Prev 2000 9 1037 1042 11045785

[B10] Sakiyama T Kohno T Mimaki S Ohta T Yanagitani N Sobue T Kunitoh H Saito R Shimizu K Hirama C Kimura J Maeno G Hirose H Eguchi T Saito D Ohki M Yokota J Association of amino acid substitution polymorphisms in DNA repair genes TP53, POLI, REV1 and LIG4 with lung cancer risk Int J Cancer 2005 114 730 737 10.1002/ijc.20790 15609317

[B11] Boldrini L Gisfredi S Ursino S Lucchi M Greco G Mussi A Fontanini G Prognostic impact of p53 Pro72 homozygous genotype in non-small cell lung cancer patients Oncol Rep 2008 19 771 773 18288414

[B12] Tommiska J Eerola H Heinonen M Salonen L Kaare M Tallila J Ristima¨ki A Von Smitten K Aittoma¨ki K Heikkila¨ P Blomqvist C Nevanlinna H Clin. Breast cancer patients with p53 Pro72 homozygous genotype have a poorer survival Clin Cancer Res 2005 11 5098 5103 10.1158/1078-0432.CCR-05-0173 16033823

[B13] Vannini I Zoli W Tesei A Rosetti M Sansone P Storci G Passardi A Massa I Ricci M Gusolfino D Fabbri F Ulivi P Brigliadori G Amadori D Bonafe M :Role of p53 codon 72 arginine allele in cell survival in vitro and in the clinical outcome of patients with advanced breast cancer Tumor Biol 2008 29 145 151 10.1159/000143400 18612219

[B14] Kim JG Sohn SK Chae YS Song HS Kwon KY Do YR Kim MK Lee KH Hyun MS Lee WS Sohn CH Jung JS Kim GC Chung HY Yu W TP53 codon 72 polymorphism associated with prognosis in patients with advanced gastric cancer treated with paclitaxel and cisplatin Cancer Chemother Pharmacol 2009 64 355 360 10.1007/s00280-008-0879-3 19052714

[B15] Zhuo XL Cai L Xiang ZL Zhuo WL Wang Y Zhang XY TP53 codon 72 polymorphism contributes to nasopharyngeal cancer susceptibility: a meta-analysis Arch Med Res 2009 40 299 305 10.1016/j.arcmed.2009.03.006 19608020

[B16] Jung IL Kang HJ Kim KC Kim IG PTEN/pAkt/p53 signaling pathway correlates with the radioresponse of non-small cell lung cancer Int J Mol Med 2010 25 517 523 20198299 10.3892/ijmm_00000372

[B17] Yip WK Leong VC Abdullah MA Yusoff S Seow HF Overexpression of phospho-Akt correlates with phosphorylation of EGF receptor, FKHR and BAD in nasopharyngeal carcinoma Oncol Rep 2008 19 319 328 18202777

[B18] Levine AJ Feng Z Mak TW You H Jin S Coordination and communication between the p53 and IGF-1-AKT-TOR signal transduction pathways Genes Dev 2006 20 267 275 10.1101/gad.1363206 16452501

[B19] Gottlieb TM Leal JF Seger R Taya Y Oren M Cross-talk between Akt, p53 and Mdm2: possible implications for the regulation of apoptosis Oncogene 2002 21 1299 1303 10.1038/sj.onc.1205181 11850850

[B20] Ogawara Y Kishishita S Obata T Isazawa Y Suzuki T Tanaka K Masuyama N Gotoh Y Akt enhances Mdm2-mediated ubiquitination and degradation of p53 J Biol Chem 2002 277 21843 21850 10.1074/jbc.M109745200 11923280

[B21] Meek DW The p53 response to DNA damage DNA Repair (Amst) 2004 3 1049 1056 10.1016/j.dnarep.2004.03.027 15279792

[B22] Boehme KA Kulikov R Blattner C p53 stabilization in response to DNA damage requires Akt/PKB and DNA-PK Proc Natl Acad Sci USA 2008 105 7785 7790 10.1073/pnas.0703423105 18505846 PMC2409394

[B23] Suvasini R Somasundaram K Essential role of PI3-kinase pathway in p53-mediated transcription: Implications in cancer chemotherapy Oncogene Oncogene 2010 29 3605 3618 10.1038/onc.2010.123 20418912

[B24] Huang WC Chen CC Akt phosphorylation of p300 at Ser-1834 is essential for its histone acetyltransferase and transcriptional activity Mol Cell Biol 2005 25 6592 6602 10.1128/MCB.25.15.6592-6602.2005 16024795 PMC1190347

[B25] Hara A Okayasu I Cyclooxygenase-2 and inducible nitric oxide synthase expression in human astrocytic gliomas: correlation with angiogenesis and prognostic significance Acta Neuropathol 2004 108 43 48 10.1007/s00401-004-0860-0 15088099

[B26] Salvioli S Bonafe M Barbi C Storci G Trapassi C Tocco F Gravina S Rossi M Tiberi L Mondello C Monti D Franceschi C p53 codon 72 alleles influence the response to anticancer drugs in cells from aged people by regulating the cell cycle inhibitor p21WAF1 Cell Cycle 2005 4 1264 1271 10.4161/cc.4.9.1978 16082224

[B27] Den Reijer PM Maier AB Westendorp RG Van Heemst D Influence of the TP53 codon 72 polymorphism on the cellular responses to X-irradiation in fibroblasts from nonagenarians Mech Ageing Dev 2008 129 175 182 10.1016/j.mad.2007.12.006 18272203

[B28] Landers JE Cassel SL George DL Translational enhancement of mdm2 oncogene expression in human tumor cells containing a stabilized wild-type p53 protein Cancer Res 1997 57 3562 3568 9270029

[B29] Bergamaschi D Gasco M Hiller L Sullivan A Syed N Trigiante G Yulug I Merlano M Numico G Comino A Attard M Reelfs O Gusterson B Bell AK Heath V Tavassoli M Farrell PJ Smith P Lu X Crook T p53 polymorphism influences response in cancer chemotherapy via modulation of p73-dependent apoptosis Cancer Cell 2003 3 387 402 10.1016/S1535-6108(03)00079-5 12726864

[B30] Marin MC Jost CA Brooks LA Irwin MS O’Nions J Tidy JA James N McGregor JM Harwood CA Yulug IG Vousden KH Allday MJ Gusterson B Ikawa S Hinds PW Crook T Kaelin WG Jr A common polymorphism acts as an intragenic modifier of mutant p53 behaviour Nat Genet 2000 25 47 54 10.1038/75586 10802655

[B31] Tada M Furuuchi K Kaneda M Matsumoto J Takahashi M Hirai A Mitsumoto Y Iggo RD Moriuchi T Inactivate the remaining p53 allele or the alternate p73? Preferential selection of the Arg72 polymorphism in cancers with recessive p53 mutants but not transdominant mutants Carcinogenesis 2001 22 515 517 10.1093/carcin/22.3.515 11238194

[B32] Lo KW Huang DP Genetic and epigenetic changes in nasopharyngeal carcinoma Semin Cancer Biol 2002 12 451 462 10.1016/S1044579X02000883 12450731

[B33] Storey A Thomas M Kalita A Harwood C Gardiol D Mantovani F Breuer J Leigh IM Matlashewski G Banks L Role of a p53 polymorphism in the development of human papilloma-virus-associated cancer Nature 1998 393 229 234 10.1038/30400 9607760

[B34] Lopez-Lizarraga E Sanchez-Corona J Montoya-Fuentes H Bravo-Cuellar A Campollo-Rivas O Lopez-Demerutis E Morgan-Villela G Arcaute-Velazquez F Monreal-Martinez JA Human papillomavirus in tonsillar and nasopharyngeal carcinoma: isolation of HPV subtype 31 Ear Nose Throat J 2000 79 942 944 11191432

[B35] Mirzamani N Salehian P Farhadi M Tehran EA Detection of EBV and HPV in nasopharyngeal carcinoma by in situ hybridization Exp Mol Pathol 2006 81 231 234 10.1016/j.yexmp.2006.04.006 16787643

[B36] Maxwell JH Kumar B Feng FY McHugh JB Cordell KG Eisbruch A Worden FP Wolf GT Prince ME Moyer JS Teknos TN Chepeha DB Stoerker J Walline H Carey TE Bradford CR HPV-positive/p16-positive/EBV-negative nasopharyngeal carcinoma in white North Americans Head Neck 2010 32 562 567 19757421 10.1002/hed.21216 PMC2855405

[B37] Faccioli S Cavicchi O Caliceti U Rinaldi Ceroni A Chieco P Cell proliferation as an independent predictor of survival for patients with advanced nasopharyngeal carcinoma Mod Pathol 1997 10 884 894 9310951

[B38] Genc E Hosal AS Gedikoglu G Ozyar E Sozeri B Prognostic value of p53, proliferating cell nuclear antigen, and Ki-67 expression in undifferentiated nasopharyngeal carcinomas Otolaryngol Head Neck Surg 2000 122 868 873 10.1016/S0194-5998(00)70016-7 10828801

[B39] Shi W Pataki I MacMillan C Pintilie M Payne D O’Sullivan B Cummings BJ Warde P Liu FF Molecular pathology parameters in human nasopharyngeal carcinoma Cancer 2002 94 1997 2006 10.1002/cncr.0679 11932902

[B40] Tsai ST Jin YT Leung HW Wang ST Tsao CJ Su IJ Bcl-2 and proliferating cell nuclear antigen (PCNA) expression correlates with subsequent local recurrence in nasopharyngeal carcinomas Anticancer Res 1998 18 2849 2854 9713473

[B41] Ma BB Poon TC To KF Zee B Mo FK Chan CM Ho S Teo PM Johnson PJ Chan AT Prognostic significance of tumor angiogenesis, Ki 67, p53 oncoprotein, epidermal growth factor receptor and HER2 receptor protein expression in undifferentiated nasopharyngeal carcinoma–a prospective study Head Neck 2003 25 864 872 10.1002/hed.10307 12966511

[B42] Masuda M Shinokuma A Hirakawa N Nakashima T Komiyama S Expression of bcl-2-, p53, and Ki-67 and outcome of patients with primary nasopharyngeal carcinomas following DNA-damaging treatment Head Neck 1998 20 640 644 10.1002/(SICI)1097-0347(199810)20:7<640::AID-HED11>3.0.CO;2-K 9744466

[B43] Wang LF Chai CY Kuo WR Tai CF Lee KW Ho KY The prognostic value of proliferating cell nuclear antigen (PCNA) and p53 protein expression in patients with advanced nasopharyngeal carcinoma Acta Otolaryngol 2006 126 769 774 10.1080/00016480500469545 16803719

[B44] Kouvidou C Stefanaki K Dai Y Tzardi M Koutsoubi K Darivianaki K P21/waf1 protein expression in nasopharyngeal carcinoma. Comparative study with PCNA, p53 and MDM-2 protein expression Anticancer Res 1997 17 2615 2619 9252690

[B45] Yang HY Wen YY Chen CH Lozano G Lee MH 14-3-3 sigma positively regulates p53 and suppresses tumor growth Mol Cell Biol 2003 23 7096 7107 10.1128/MCB.23.20.7096-7107.2003 14517281 PMC230310

[B46] Yang H Zhao R Lee MH 14-3-3sigma, a p53 regulator, suppresses tumor growth of nasopharyngeal carcinoma Mol Cancer Ther 2006 5 253 860 16505098 10.1158/1535-7163.MCT-05-0395

[B47] Yi B Tan SX Tang CE Huang WG Cheng AL Li C Zhang PF Li MY Li JL Yi H Peng F Chen ZC Xiao ZQ Inactivation of 14-3-3 sigma by promoter methylation correlates with metastasis in nasopharyngeal carcinoma J Cell Biochem 2009 106 858 866 10.1002/jcb.22051 19160382

[B48] Schuurbiers OC Kaanders JH van der Heijden HF Dekhuijzen RP Oyen WJ Bussink J The PI3-K/AKT-pathway and radiation resistance mechanisms in non-small cell lung cancer J Thorac Oncol 2009 4 761 767 10.1097/JTO.0b013e3181a1084f 19404218

[B49] Bode AM Dong Z Post-translational modification of p53 in tumorigenesis Nat Rev Cancer 2004 4 793 805 10.1038/nrc1455 15510160

[B50] Ozeki C Sawai Y Shibata T Kohno T Okamoto K Yokota J Tashiro F Tanuma S Sakai R Kawase T Kitabayashi I Taya Y Ohki R Cancer susceptibility polymorphism of p53 at codon 72 affects phosphorylation and degradation of p53 protein J Biol Chem 2011 286 18251 18260 10.1074/jbc.M110.208587 21454683 PMC3093897

